# Anion-Regulated Synthesis of ZnO 1D Necklace-Like Nanostructures with High Photocatalytic Activity

**DOI:** 10.1186/s11671-020-03435-5

**Published:** 2020-11-04

**Authors:** Xiaoyun Qin, Dongdong Shi, Bowen Guo, Cuicui Fu, Jin Zhang, Qingqing Xie, Xiangdong Shi, Fenghua Chen, Xiaomei Qin, Wei Yu, Xiangli Feng, Yan Liu, Dan Luo

**Affiliations:** 1 School of Material and Chemical Engineering, Zhengzhou University of Light Industry, Zhengzhou, 450002 China; 2 State Key Laboratory of Heavy Oil Processing, College of New Energy and Materials, Beijing Key Laboratory of Biogas Upgrading Utilization, China University of Petroleum Beijing, Beijing, 102249 China; 3 Laboratory of Biomimetic Nanomaterials, Department of Orthodontics, Peking University School and Hospital of Stomatology, National Engineering Laboratory for Digital and Material Technology of Stomatology, Beijing Key Laboratory of Digital Stomatology, Beijing, 100081 China; 4 Key Lab of Separation Science for Analytical Chemistry, Dalian Institute of Chemical Physics, Chinese Academy of Sciences, Dalian, 116023 Liaoning China; 5 Department of Stomatology, Hubei Provincial Hospital of Traditional Chinese Medicine, Wuhan, 430065 China

**Keywords:** Anions-regulated, One-dimensional, ZnO nanostructures, Photocatalyst

## Abstract

One-dimensional (1D) nanomaterials with specific architectures have received increasing attention for both scientific and technological interests for their applications in catalysis, sensing, and energy conversion, etc. However, the development of an operable and simple method for the fabrication of 1D nanostructures remains a challenge. In this work, we developed an “anion-regulated morphology” strategy, in which anions could regulate the dimensionally-restricted anisotropic growth of ZnO nanomaterials by adjusting the surface energy of different growth facets. ZnO 1D necklace-like nanostructures (NNS) could be prepared through a hydrothermal treatment of zinc acetate and urea mixture together with a subsequent calcination procedure at 400 °C. While replacing the acetate ions to nitrate, sulfate, and chlorion ions produced ZnO nanoflowers, nanosheets and hexagonal nanoplates, respectively. Density functional theory calculations were carried out to explain the mechanism behind the anions-regulating anisotropic crystal growth. The specified ZnO 1D NNS offered improved electron transport while the grain surface could supply enlarged specific surface area, thus providing advanced photocatalytic ability in the following photodegradation of methyl orange (MO). Among the four photocatalysts with different morphologies, ZnO 1D NNS, possessing the highest catalytic activity, degraded 57.29% MO in the photocatalytic reaction, which was 2 times, 10 times and 17 times higher than nanoflowers, nanosheets and hexagonal nanoplates, respectively. Our work provides new ideas for the construction and application of ZnO 1D nanomaterials.

## Introduction

The discovery of carbon nanotubes has triggered a vast research interest in one-dimensional (1D) nanomaterials for their efficient transport of electrons and excitons. Various 1D nanostructures, including nanowires, nanofibers, nanorods, nanobelts and nanotubes have been produced as primary building blocks for constructing the next-generation of high-performance nanodevices in studying transport processes in the one-dimensionally confined objects [1]. The specific micromorphology induces the fundamental studies in mesoscopic physics and technological applications. Throughout the 1D nanomaterials, carbon group [2, 3], III–V group [4], II–VI group, and oxide group materials [5, 6] are hot fields to be investigated and synthesized. In particular, the 1D nanoscale metal oxide semiconductors have attracted considerable attention within the past decades owing to their dimensionality-dependent physical properties in fabricating high performance electronic, magnetic, and optoelectronic devices.

Among the 1D nanomaterials, ZnO 1D nanostructures have been extensively exploited for their potential applications in optoelectronic nanodevices, piezoelectric nanogenerators, sensors, and solar cells [7–9]. As a nontoxic n-type semiconductor, ZnO has been named as one of the most important functional oxides for its wide band gap (3.37 eV) and large exciton binding energy (60 meV) [10]. Furthermore, extensive research has been focused on fabricating ZnO 1D nanostructures in correlating the dimensional morphology with the specific size-related optical and electrical properties. Particularly, Wang et al. discovered the piezoelectricity and expanded a range of applications of ZnO 1D in clean and renewable energy. The ZnO 1D nanostructures have been endowed as important as carbon nanotubes and silicon nanowires, owing to the extensive efforts in nanopiezotronics [11]. The anisotropic growth of 1D nanocrystal demands sophisticated regulation of synthetic routes to suppress the three- or two-dimensional extension. Strategies including physical vapor deposition, electrochemical deposition and template growth method, have been successfully applied for producing ZnO 1D nanostructures [7, 12, 13]. However, a simple and efficient method to accurately control the morphology of ZnO is still lacking. From the point of view of industrialization, the wet chemical method is regarded as a prospective route for mass production owing to its mild reaction conditions, less energy consuming, inexpensive equipment and simple procedure. During the commendable hydrothermal procedure, different parameters such as zinc precursor, solvents, pH, and additives all play important roles in regulating the micromorphology and even physical character of the final ZnO nanostructures [14–17]. It would be very attractive to find a simple variable based on hydrothermal method to achieve the induction of one-dimensional structure.

In this paper, we developed an “anion-regulated morphology” synthetic strategy. The ZnO 1D necklace-like nanostructure (NNS) and other three morphologies (nanoflowers, nanoflakes, and nanoplates) were easily synthesized by introducing different anions in the simple hydrothermal procedure. The subsequent calcination process was carried out to transfer the intermediate to desired oxide product. Density functional theory (DFT) calculations exhibited that acetate ions could lead to a lower surface energy of (101) facet compared to (001) facet, inducing the axial growth of ZnO crystal along the (101) plane. The growth mechanism of ZnO nanostructures were further investigated through a series of characterizations, giving a comprehensive understanding of the morphologies derived from the regulation of different anions. The acquired ZnO 1D NNS, with interconnected particle morphology, possessed a larger surface area and a higher interface charge transfer rate, which facilitated surface attachment and rapid degradation of organic dyes. The NNS exhibited about 2 times, 10 times and 17 times higher than ZnO nanoflowers, nanosheets and hexagonal nanoplates in the photocatalytic degradation of methyl orange (MO).

## Experimental Methods

### Preparation of ZnO Photocatalysts

Zinc acetate dihydrate, zinc nitrate hexahydrate, zinc chloride, zinc sulfate monohydrate, urea, methyl orange (MO), disodium ethylenediaminetetraacetate (EDTA-2Na), 1,4-benzoquinone (BQ), and isopropyl alcohol (IPA) were purchased from Aladdin Ltd. (Shanghai, China). All chemicals were used as received without further purification. The water used throughout all experiments was purified through a Millipore system. In a typical experiment, 25 mL of 0.2 M zinc acetate was added into 25 mL of 0.2 M urea aqueous solution under stirring (500 rpm). The mixture was sealed in a Teflon-lined autoclave and heated at 95 °C for 6 h then cooled down to room temperature naturally. The resulting precipitate was centrifuged and washed repeatedly with deionized water and alcohol, which was then dried at 80 ℃ in an oven. Finally, ZnO 1D NNS were obtained after a calcination process at 400 °C for 0.5 h in a muffle furnace. ZnO nanflowers, irregular nanoflakes, and hexagonal nanoplates were synthesized using the same process as the above, while the only difference was that 0.2 M zinc acetate solution was altered to 0.2 M zinc nitrate, zinc chloride, and zinc sulfate solution, respectively.

### Characterization

An environmental JEOL Field-emission SU-8010 scanning electron microscope (SEM) at an accelerating applied potential of 5 kV were employed for the imaging measurements reported here. The sample for SEM characterization was prepared by placing a drop of the dispersion on a bare silicon substrate and dried under vacuum at room temperature. Transmission electron microscopy (TEM) and high-resolution TEM (HRTEM) images were recorded on a JEOL JEM 2100 transmission electron microscope operating at an accelerating voltage of 200 kV. X-ray powder diffraction (XRD) was carried out using a Rigaku Dmax-2500 X-ray diffractometer with Cu Ka radiation (*λ* = 1.54 Å) at 50 kV and 200 mA at a scanning rate of 5° min^−1^. The Brunauer–Emmett–Teller (BET) surface area and Barrett–Joyner–Halenda pore size were measured by N_2_ adsorption/desorption at 77 K using a QuadraSorb SI surface area analyzer after degassing the samples at 100 °C for 10 h. UV–Vis spectra were obtained on a UV-1800 Spectrophotometer (Shimadzu, Japan). The total organic carbon (TOC) analyzer (Multi N/C 2100, Analytik Jena AG) was applied to analyse the mineralization degree of MO solution.

### Density Functional Theory (DFT) Calculations

The DFT calculations were performed by the Dmol^3^ module in Materials Studio 5.5 package. Ultra-soft pseudopotential method was applied while the Generalized gradient approximation (GGA) in the scheme of PW91 was employed in the description of exchange–correlation interaction. The energy cutoff was set to 400 eV, and the convergence of energy was set to be 10^–4^ eV while that of force was 10^–2^ eV/Å. All of the models were calculated in periodically boxes with a vacuum slab of 30 Å to separate the interaction between periodic images. The simulated unit cell was 3.249 × 3.249 × 5.207 Å ^3^.

The difference of facet energy (Δ*E*) per ZnO unit is defined as,

$$\Delta E = E_{{{\text{facet}} + {\text{ligand}}}} - E_{{{\text{facet}}}} - E_{{{\text{ligand}}}}$$

where *E*_facet+ligand_ is the total energy of a given facet and one ligand molecule binding on its surface per supercell, *E*_facet_ is the energy of the facet, and *E*_ligand_ is the energy of the adding anions. (The negative sign indicates the favorable of binding interaction.)

### Photocatalytic Experiments

The photocatalytic performance of the ZnO photocatalyst was evaluated under identical conditions using a representative dye MO under UV-light irradiation in an immersion well photochemical reactor made of Pyrex glass, which is equipped with a magnetic stirring bar, a water circulating jacket and an opening to supply air. For each experiment, 10 mg of ZnO photocatalyst was dispersed in 50 mL of 10 mg/L of the MO aqueous solution. A 6 W UV lamp of wavelength 365 nm was employed as light source. The distance between the light source and surface of the solution was 6 cm. Prior to irradiation, the suspension was stirred in the dark for 30 min to ensure an adsorption/desorption equilibrium between the catalyst and organic dyes. 1 mL of aliquots were withdrawn at different time intervals, centrifuged and analyzed by recording variations in the UV–Vis absorption spectra. All experiments were repeated at least three times and the mean value is reported along with the standard deviation. According to the Beer's law, the UV–Vis absorption curves of MO aqueous solution from 2 to 10 mg L^−1^ were recorded to give a linear relationship as *A* = 0.068 *C*_MO_, suggesting the residual concentration of MO (*C*_MO_) and degradation rate (*R*) can be calculated from the absorbance (*A*). The degradation rate of MO dye was estimated using the following equation,

$$R = \frac{{\left( {C_{0} - C_{\text{t}} } \right)}}{{C_{0} }} \times 100\%$$

where *C*_0_ is the initial concentration of dye and *C*_t_ is the concentration of the dye after irradiation time *t*. After the degradation experiments, the ZnO catalyst was separated from the reaction mixture and washed and dried to carry out the reusability tests.

## Results and Discussion

### Characterization of Catalysts

**Scheme 1:**
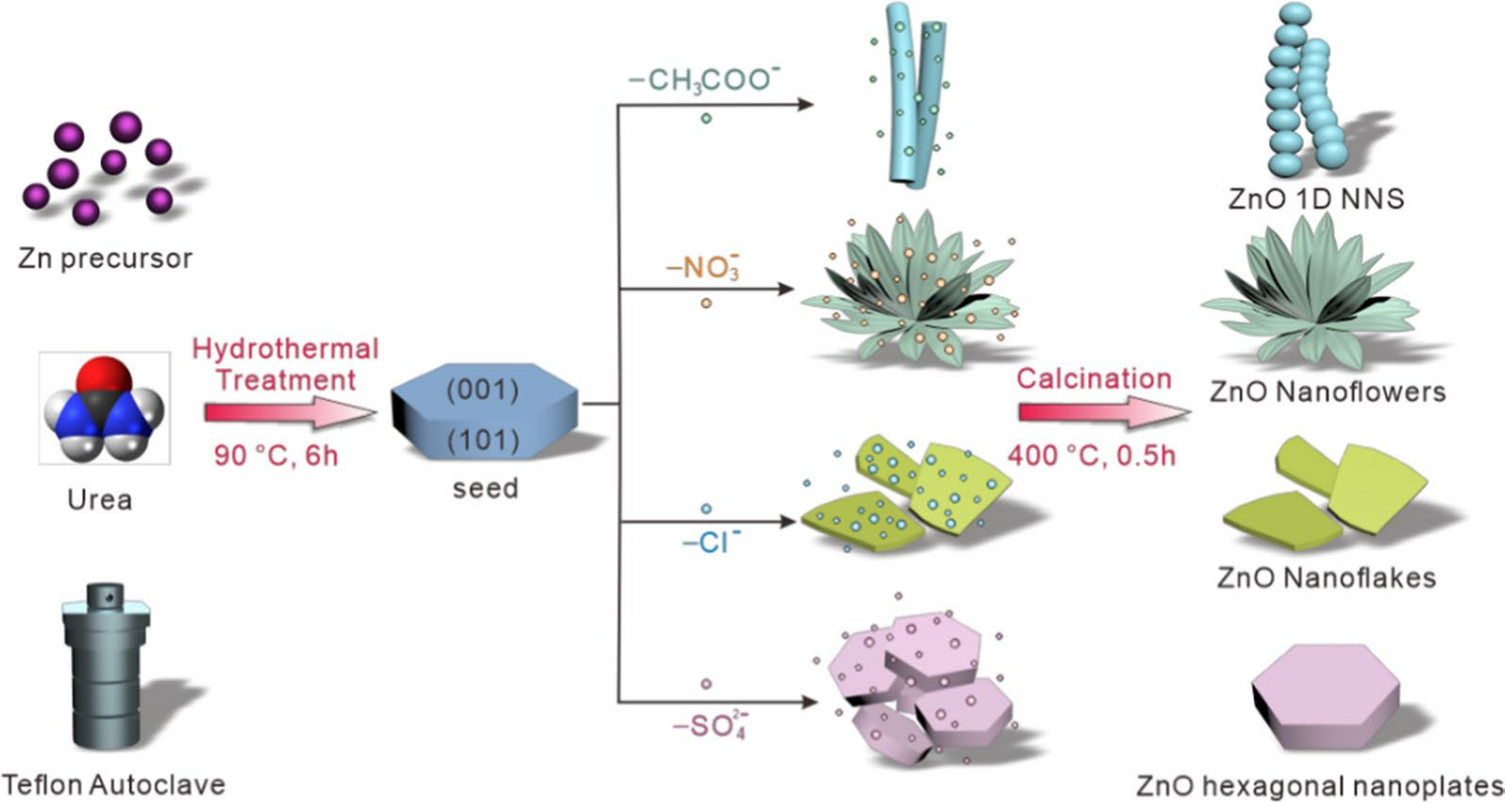
Schematic description of the formation of ZnO 1D NNS, nanoflowers, nanoflakes, and hexagonal nanoplates in the presence of acetate, nitrate, chloride, and sulfate anions

Scheme 1 describes the preparation procedures of ZnO nanostructures by adding different zinc precursors and urea into the Teflon autoclave undergoing a hydrothermal treatment at 90 °C for 6 h. The resulting suspension was centrifuged and then experienced a calcination procedure under air atmosphere to transfer the intermedium to oxide products. The preparation of ZnO nanostructures consisted of the chemical reactions as follows [18],

1 $${\text{CO}}\left( {{\text{NH}}_{{2}} } \right)_{{2}} + {\text{H}}_{{2}} {\text{O}} \to {\text{CO}}_{{2}} \uparrow + {\text{2NH}}_{{3}} \uparrow$$

2 $${\text{NH}}_{{3}} + {\text{H}}_{{2}} {\text{O}} \rightleftharpoons {\text{NH}}_{{3}} \cdot {\text{H}}_{{2}} {\text{O}} \rightleftharpoons {\text{NH}}_{{4}}^{ + } + {\text{OH}}^{ - }$$

3 $${\text{Zn}}^{{{2} + }} + {\text{4NH}}_{{3}} \to {\text{ Zn}}\left( {{\text{NH}}_{{3}} } \right)_{{4}}^{{{2} + }}$$

4 $${\text{Zn}}\left( {{\text{NH}}_{{3}} } \right)_{{4}}^{{{2} + }} + {\text{2OH}}^{ - } \to {\text{ ZnO}} \downarrow + {\text{4NH}}_{{3}} \uparrow + {\text{H}}_{{2}} {\text{O}}$$

urea hydrolyzed in water to release alkaline ammonia (Eq. 1), which then formed a weak base in water solution (Eq. 2). The addition of zinc ions promoted the formation of tetrahedron Zn(NH_3_)_4_^2+^ (Eq. 3) and nucleation of ZnO seed crystal (Eq. 4). Finally, ZnO nanostructures were produced by the consequent calcination procedure under air. The four kinds of anions seem to absorb on the intersecting facets of seed crystal and bring out inverse growth tendency. The growth procedure and mechanism of ZnO nanostructures were surveyed through a series of characterizations to give a comprehensive understanding of the morphologies originating from different anions.

**Fig. 1:**
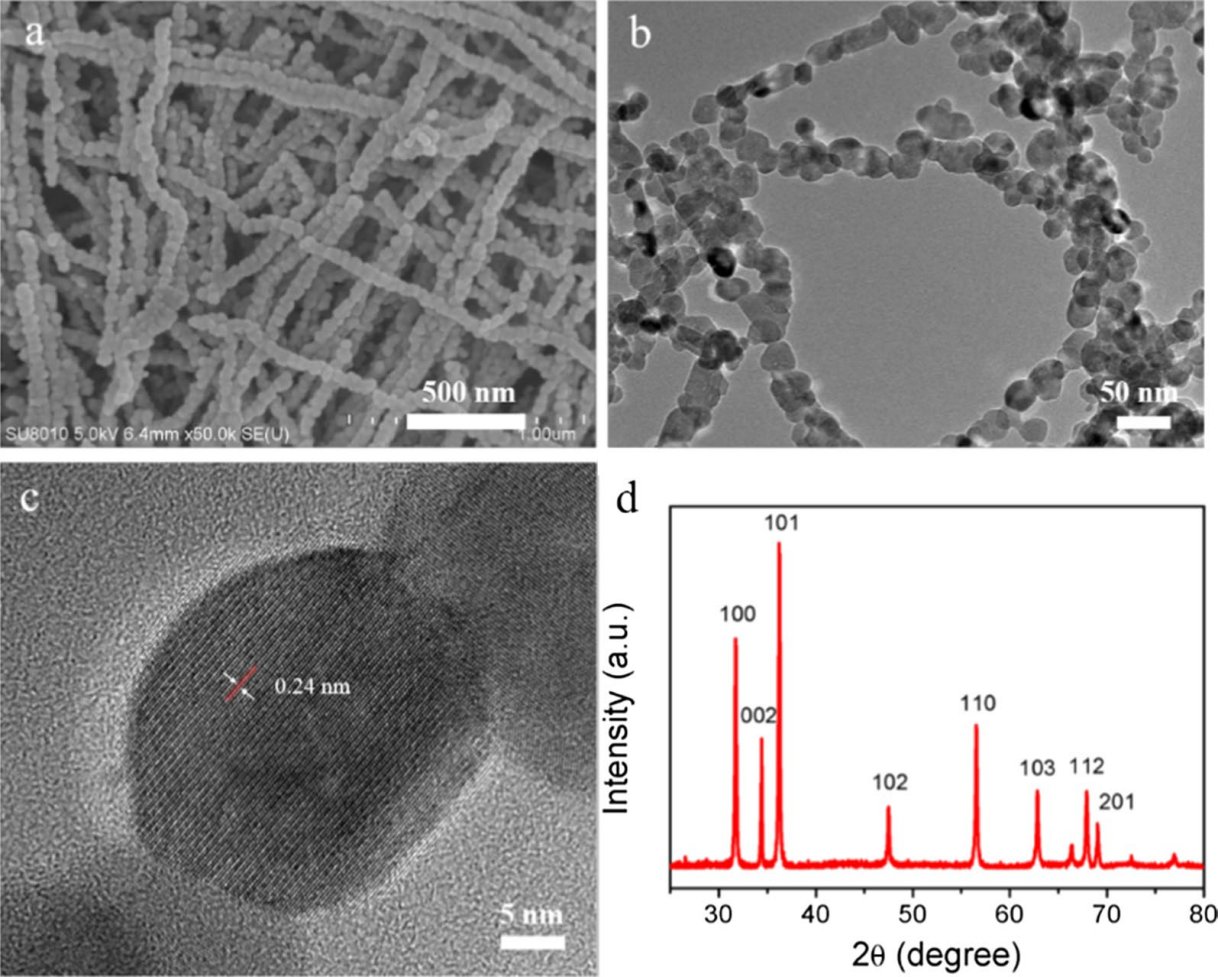
Morphological and structural analysis of ZnO 1D NNS. **a** SEM, **b** TEM, **c** HR-TEM image and XRD pattern of ZnO 1D NNS

Figure 1a shows the micromorphology of the prepared ZnO 1D NNS. A mass of string of beads with diameter around 30–50 nm were obtained after the hydrothermal and calcination procedure. The uniform ZnO beads bunched to form rough lines about several micrometers in length. The ZnO nanoparticles can be clearly distinguished in the TEM image as shown in Fig. 1b. The HR-TEM image of an individual nanoparticle revealed parallel lattice fringes with assessed interplanar spacing of 0.24 nm, corresponding to (101) lattice space of ZnO (Fig. 1c) [19, 20]. XRD profile of the as-synthesized samples is shown in Fig. 1d. The presence of all characteristic peaks confirms the successful preparation of hexagonal wurtzite ZnO phase (JCPDS Card No. 36-1451) in the synthesized procedure.

**Fig. 2:**
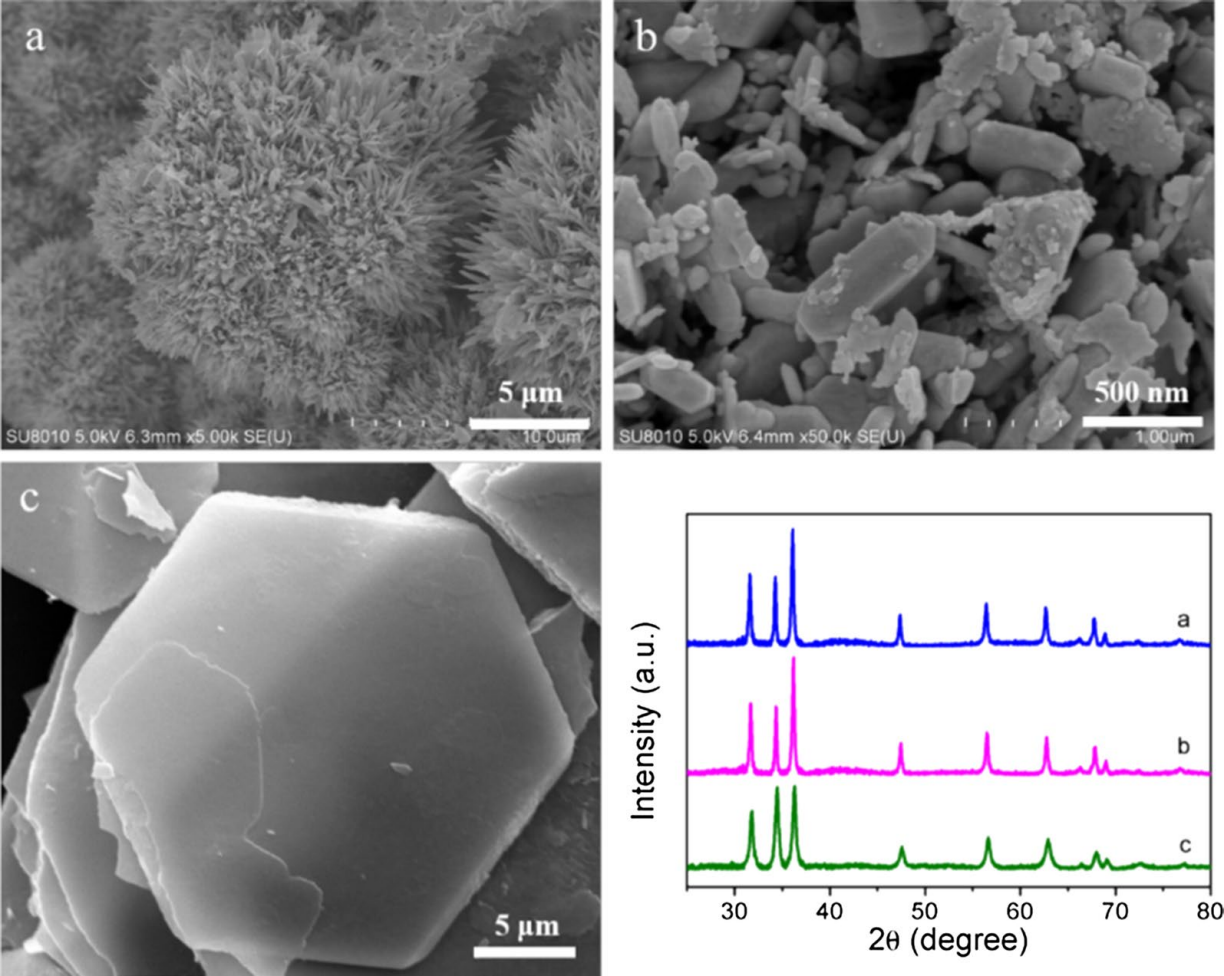
Morphological and structural analysis of other ZnO nanostructures obtained by using zinc nitrate, zinc sulfate, and zinc chlorion as precursor. SEM images of **a** ZnO nanoflowers, **b** nanoflakes, **c** hexagonal nanoplates, and **d** their corresponding XRD patterns

Changing the zinc precursor by introducing different anions into the growth medium engineered different types and dimensions of ZnO nanostructures grown under the same conditions. Zinc nitrate, zinc chloride, and zinc sulfate were introduced to obtain other micromorphologies as shown in Fig. 2a–c, respectively. The nanoflowers was aggregated by narrow pieces with a sharp tip and a ~ 5 μm length, while the ZnO product grown under the effect of nitrate anions in the growth solution. A mass of irregular nanoflakes with ~ 100 nm thickness were observed when zinc chloride served as precursor in the hydrothermal procedure. While adding zinc sulfate would generate hexagonal nanoplates with diameter in ~ 25 μm and thickness in several hundred nanometers. Figure 2d shows the XRD patterns of the three ZnO nanostructures, exhibiting identical peak positions with the ZnO 1D NNS. These data suggest that all the ZnO nanostructures can be indexed to hexagonal wurtzite ZnO phase [21]. The UV–Vis spectra of the four ZnO nanostrucutres is shown in Additional file 1: Fig. S1, exhibiting little difference between different micromophologies. Absorption edge at around 370 nm can be attributed to the electronic transition of ZnO from valence band (VB) to conduction band (CB), which is in accordance with previous report [20]. The change in micromophologies would not shift the band edge absorption of ZnO.

### Growth Mechanism Under Different Anions

**Fig. 3:**
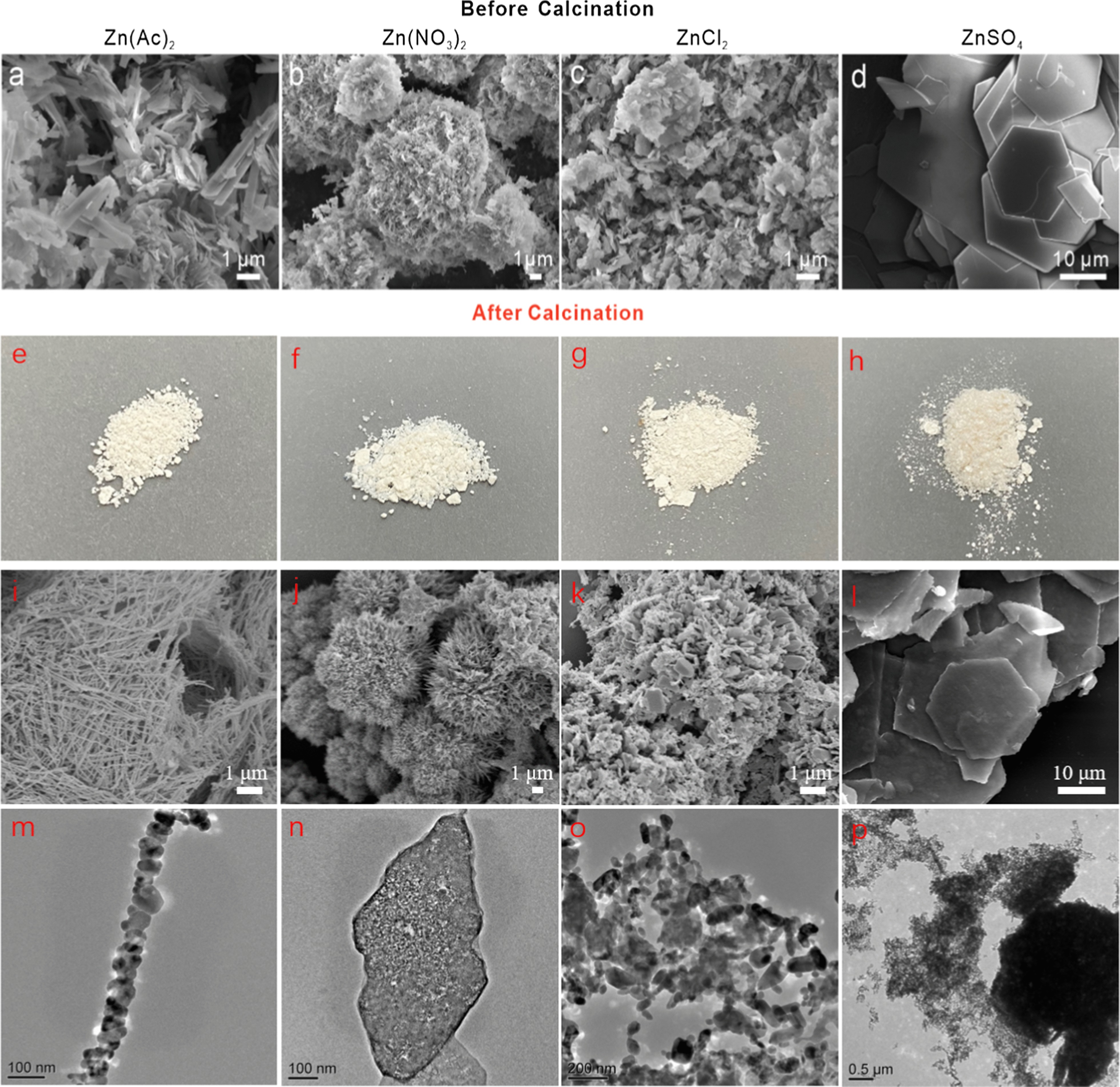
SEM images, digital photographs, and TEM images of ZnO nanostructures using **a**,** e**,** i**,** m** acetate, **b**,** f**,** j**,** n** nitrate, **c**,** g**,** k**,** o** chloride, and **d**,** h**,** l**,** p** sulfate anions as zinc precursor before and after the calcination procedure

It has been reported that the wurtzite ZnO is crystallized by stacking alternatively zinc plane and oxygen plane along the *c*-axis [21]. Structurally, the ZnO grain crystallizes in three directions as following: a top polar zinc (001) plane, six symmetric nonpolar (101) planes normal to the former direction, and a basal polar oxygen (00$\stackrel{-}{1}$) face [17, 22]. The zinc terminated (001) plane ranks the highest surface energy compared with the side nonpolar (101) faces, inducing the lowest extension of this plane and ZnO 1D growing along (001) is energetically preferred for the total energy minimization. Figure 3 exhibits the SEM images of the four products before and after the calcination procedure while using the four kinds of anions in the zinc precursor. It can be observed that the subsequent calcination makes the ZnO nanostructures partly decompose as the gas releasing of NH_3_, CO_2_, H_2_O from the architecture and transform to porous 1D NNS, nanoflowers, nanoflakes, and hexagonal nanoplates with rough surface and smaller size. ZnO 1D nanostructures with large aspect ratio are readily produced after the hydrothermal procedure in the presence of acetate anions (Fig. 3a). Yang et al*.* have synthesized corn-like ZnO nanorods using zinc acetate as a zinc precursor and oxalic acid as a precipitator [23]. It seems like that the anions with big molecular volume prone to product 1D nanostructures preferentially. Zinc acetylacetonate was exploited as the zinc precursor to verify the inference and 1D nanorods were obtained as shown in Additional file 1: Fig. S2. The big anions tend to induce the assembling of ZnO crystalline grains along *c*-axis. These huge anions can chelate the zinc ions to form molecular plane that stacks with each other due to hydrogen bond. The growth across the molecule planes has been heavily hindered while the perpendicular growth develops along the connecting hydrogen bonds [23]. Consequently, the crystal nucleus grow along the *c*-axis due to the preferential growth of (101) facet to minimum the surface energy. 1D anions-chelating ZnO architectures are formed by hydrogen bond under hydrothermal condition. The subsequent calcination makes the 1D nanostructures partly decompose and transform to a porous necklace-like nanostructure, as compared in Fig. 3a, i, m. The similar degassing process occurred in the condition of other three anions participating in the reaction. Moreover, Additional file 1: Fig. S3 gives a detailed surface topography with grainy surface of ZnO nanoflowers when using nitrate anions in the hydrothermal treatment.

In comparison to the homogeneous 1D structure in the presence of acetate anions, the tapering agave-like ZnO pieces are clustered to form nanoflowers when zinc nitrate served as a precursor (Fig. 3b, j, n). It is worth noting that the growth rate of ZnO in the presence of nitrate is much faster than that in acetate anions, since zinc ions are gradually released in the latter due to weak ionization nature of acetate anions. It is presumed that the agave-like ZnO pieces are individually grown from spontaneous nucleation, which then forms the final bushlike aggregates [24]. The precondition that growth rate exceeded the diffusion rate of zinc cations would encounter an inhomogeneous growth in the 1D nanostructure, resulting in the tapering gradient of ZnO pieces from base to tip. When the anion was replaced as chlorion, its preferential electrostatic adsorption on the positively charged zinc cations terminated (001) plane is predominant, due to the extremely small size and thus decreases the (001) surface energy [25, 26]. That is why the irregular nanoflakes were produced as an increased growth rate for (001) plane facilitating the formation of planar morphology (Fig. 3c, k, o). It has been reported that sulfate anions could also block the active sites of ZnO surface inducing the formation of 2D structure, such as the hexagonal nanoplates shown in Fig. 3d, l, p [15, 20]. The growth rate of the polar facets is blocked, resulting in that the other facets appear with a high-index. The consequence of continuous growth in the six directions led to the formation of hexagonal nanoplates.

**Fig. 4:**
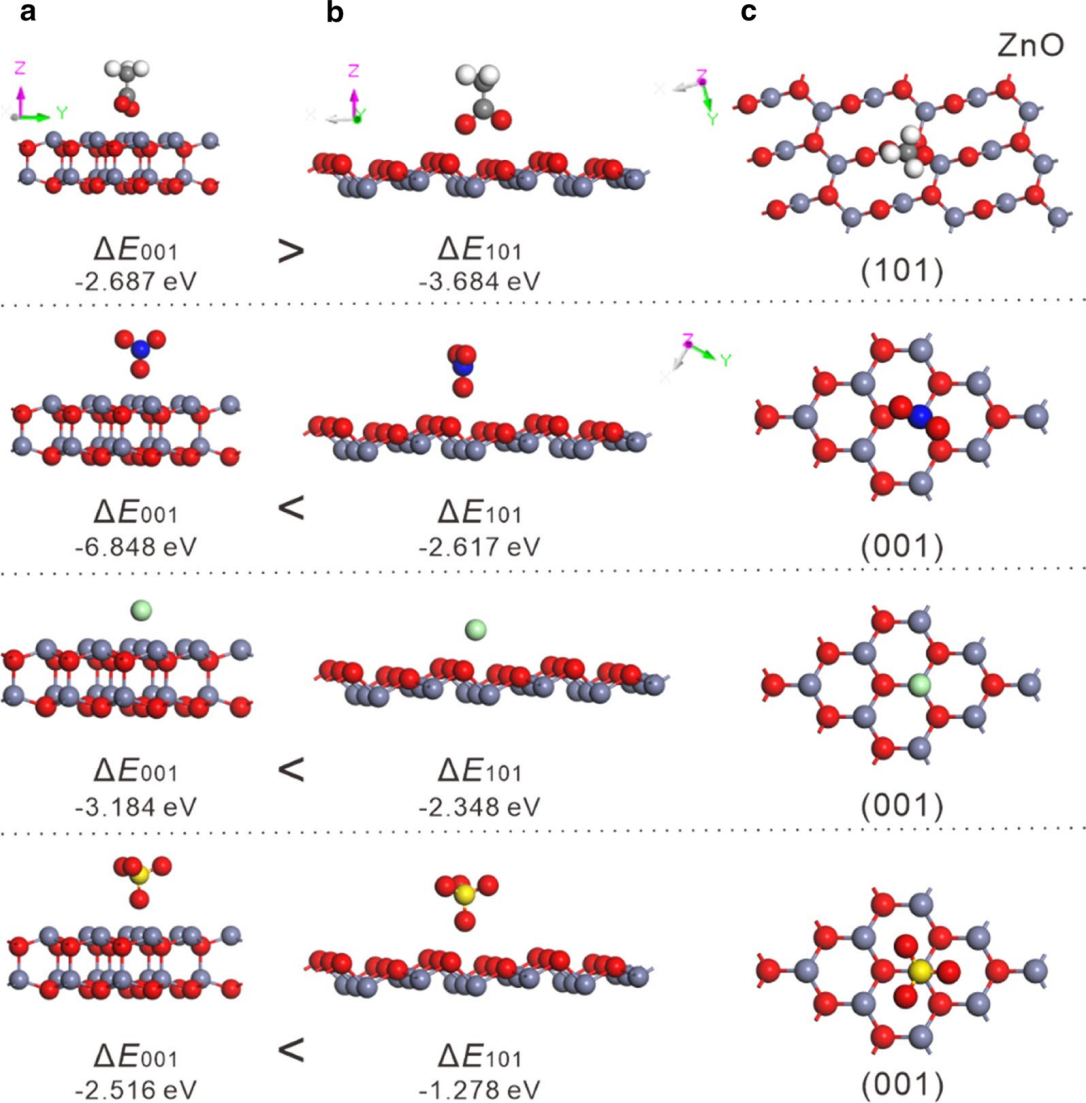
Side view of the optimized structures of anions adsorbed on **a** (001) facet and **b** (101) facet. **c** Top view of the optimized structures of anions adsorbed on (001) facet and (101) facet. The diagram illustrates the calculated Δ*E* before and after anion binding. Anions from top to down: acetate ion (CH_3_COO^−^), nitrate ion (NO_3_^−^), chloride ion (Cl^−^), and sulfate ion (SO_4_^2−^). Blue gray, Zn; Red, O; Gray, C; White, H; Blue, N; Green, Cl; Yellow, S atom

To explore the reaction mechanism, DFT calculation was used to investigate the influence of different anions on the energy of ZnO (001) and (101) facets [27–29]. Figure 4 lists the difference of facet energy (Δ*E*) before and after anion binding. The results showed that the acetate could reduce more energy in (101) facet (− 3.684 eV) than in (001) facet (− 2.687 eV), suggesting that the growth of zinc oxide (101) facet is more favorable, and the extension of (001) plane is suppressed. On the contrary, Δ*E* on (001) facets is significantly lower than that of (101) facets in presence of nitrate, chloride and sulfate anions, promoting the growth of (001) plane and finally forming flake-like morphology.

### Photocatalytic Activity

In order to investigate the photocatalytic properties of the ZnO 1D NNS, nanoflowers, irregular nanoflakes, and hexagonal nanoplates, nitrogen adsorption–desorption tests were carried out to survey the surface area and pore size of the above nanostructures. These parameters were important factors contributing to advanced photocatalytic activity for providing more active sites to adsorb pollutants on the surface of catalyst. Table 1 shows the above data, suggesting the porous nature of the prepared materials. The calculated surface area of ZnO 1D NNS, nanoflowers, nanoflakes, and hexagonal nanoplates were 60.3, 33.5, 22.9, and 15.8 m^2^ g^−1^, respectively. The relative pore volume of various ZnO nanostrucutres were measured to be 0.156, 0.106, 0.064, and 0.036 m^3^ g^−1^. These data suggest the potential superiority of ZnO 1D NNS in the following photocatalytic events compared with the other nanostructures. Photocatalytic properties for the different ZnO nanostructures were evaluated by the decomposition of an anionic dye MO. The UV–Vis absorption curves of MO aqueous solution from low to high concentration were recorded to obtain the most appropriate wavelength to monitor the photocatalytic degradation process. From Additional file 1: Fig. S4, we observed that the maximum absorbance of all the curves occurred at 465 nm, indicating of the minimum error at this position. Therefore, the absorbance at 465 nm was taken as characteristic adsorption in the photodegradation of MO. According to Beer's law, a linear relationship is established from 2 to 10 mg L^−1^ as *A* = 0.068 *C*_MO_, suggesting the residual concentration of MO and degradation rate can be calculated from the equation.

**Table 1:** The surface area and pore size of the four kinds of ZnO nanostrucutres

Sample	Zinc precursor	Surface area (m^2^ g^−1^)	Pore size (m^3^ g^−1^)
ZnO 1D NNS	Zinc acetate	60.3	0.156
ZnO nanoflowers	Zinc nitrate	33.5	0.106
ZnO nanoflakes	Zinc chloride	22.9	0.064
ZnO nanoplates	Zinc sulfate	15.8	0.036

**Fig. 5:**
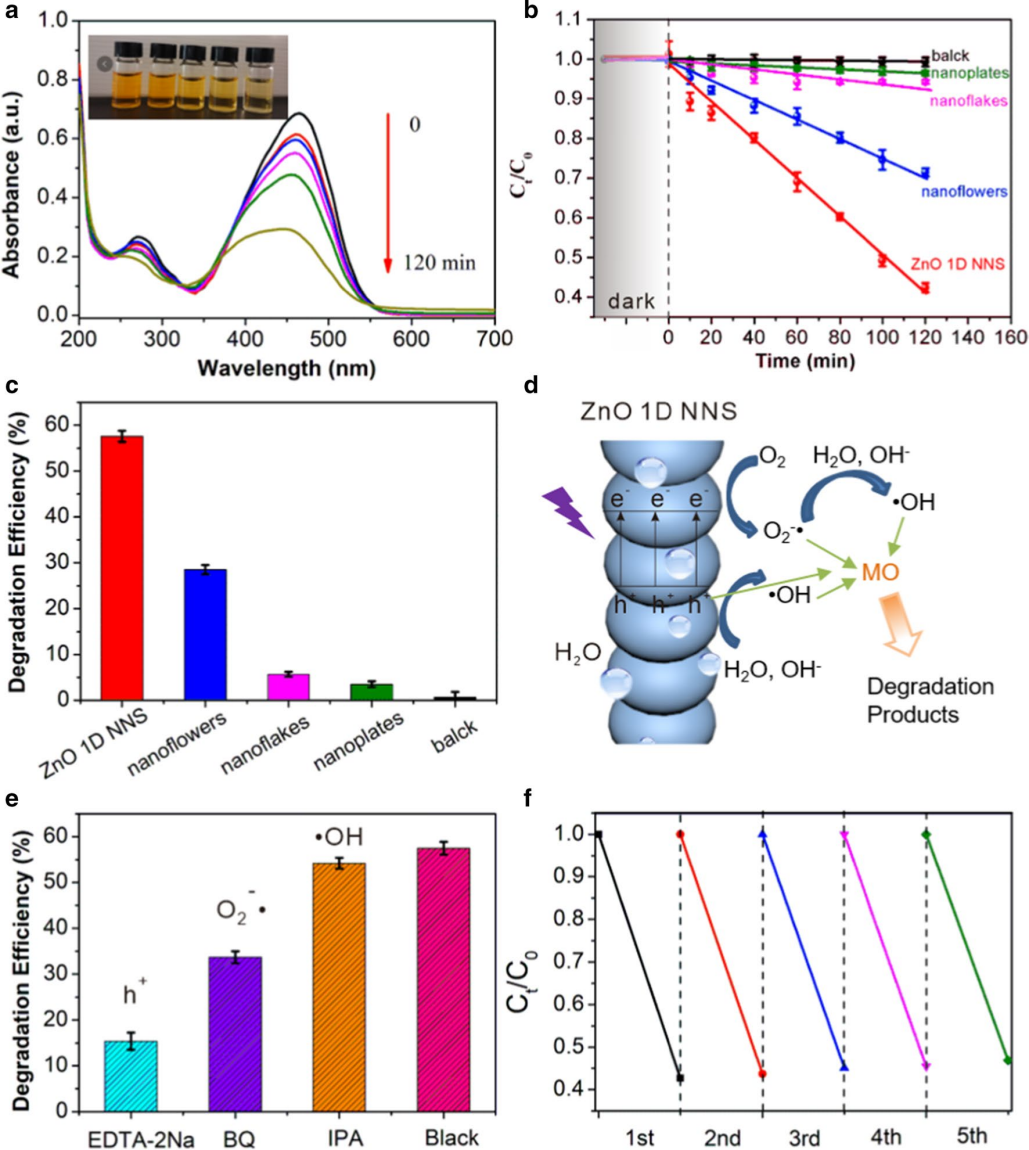
The study of photocatalytic performance of ZnO catalyst. **a** Photodegradation curves of MO solution by using ZnO 1D NNS as catalyst under irradiation with UV light. Inset shows the optical photographs of MO solution along the UV irradiation. **b** The kinetic relationship of *C*_t_/*C*_0_ versus irradiation time curves. **c** The calculated degradation efficiency by using different ZnO nanostructures. **d** Schematic representation of photocatalytic process. **e** The degradation efficiency in the presence of different radical scavengers. **f** The reuse activity of ZnO 1D NNS for the photodegradation of MO

Figure 5a shows the change in absorption intensity at different time intervals under UV irradiation (365 nm) of MO aqueous solution in the presence of ZnO 1D NNS, whereas the inset records the decolorization during the exposure. By irradiation with UV light, the characteristic absorption of MO at 465 nm decreased gradually for losing of chromophoric groups. Furthermore, the gradual blueshift of the absorption peak explained the destruction of *π*-structure during the photoinduced decomposition of MO molecule. Figure 5b plots the photodegradation dynamic curves in the presence of the four different ZnO nanostrucutres at a fixed dye concentration (10 mg L^−1^) and catalyst loading (0.2 g L^−1^). Control experiments were carried out in the absence of ZnO catalyst as well, exhibiting ignorable decoloration ratio of MO during the 2 h’ irradiation. The photocatalytic performances of ZnO with different microstructures consequently had different performance in photodegradation of MO. However, the ZnO nanomaterials exhibited analogous inaction during the adsorption–desorption period before irradiation to establish equilibrium on the material surface. This finding indicates the adsorption of stain on the catalyst surface was too limited to eliminate the dye in the medium. Though the ZnO 1D NNS had the largest surface area and pore volume, no obvious improvement was observed during the dark procedure. It may indicate that the limited improvement in adsorption capacity cannot be responsible for the complete decomposition of MO. The degradation curve could be fitted reasonably well by a pseudo-first-order kinetics equation of − ln(*C*_t_/*C*_0_) = *k*t, where *k* is the apparent pseudo-first-order rate constant (min^−1^), *C*_0_ and *C*_t_ are the MO concentration at initial and time *t*, respectively. The calculated values of *k* were used to evaluate the rate of photocatalytic reaction. The photodegradation rate of MO with ZnO 1D NNS, nanoflowers, irregular nanoflakes, and hexagonal nanoplates under UV light irradiation were calculated as 57.29%, 28.34%, 5.79%, and 3.40%, respectively (Fig. 5c). The ZnO 1D NNS sample equipped the highest activity of ~ 2 times higher than the second place in the photocatalytic events. TOC analyzer was used to evaluate the degree of mineralization of MO under optimized experimental conditions for ZnO photocatalysts. The TOC removal is calculated as 25.3%, exhibiting that the discolored MO molecules are partly mineralized to CO_2_ and H_2_O during the photodegradation in the presence of ZnO 1D NNS photocatalyst. The much lower activities of other ZnO nanostrucutres were related to the higher resistivity, due to lower mobility with charge carrier scattering, while the ZnO 1D NNS benefited from the one-dimensional construction exhibiting increased conductivity and transparency. It is worth noting that the small dose of catalyst was added to insure the effective degradation efficiency and enough high transparency in the MO photodegradation system. The resulting absorption of incident light rapidly excited abundant free electrons and holes to harvest enhanced conductivity. It is found that the ZnO 1D NNS showed higher photocatalytic activity as compared to nanflowers, irregular nanoflakes, and hexagonal nanoplates. The orientated morphological surface of ZnO 1D NNS may delay in recombination of electron–hole pairs and enhance the adsorption of oxygen on space charge region along the longitudinal direction of the interconnected ZnO 1D NNS [13, 17]. The enhanced oxygen adsorption on the surface of ZnO 1D NNS decreased the recombination of carriers by accepting photogenerated holes. In this case, the reactive oxygen species were formed to finally enhance the photocatalytic activity. Therefore, the formation rate of hydroxyl radical along the longitudinal surface of ZnO 1D NNS was higher, compared to other nanostructures [30]. It has been reported that the (101) facet of ZnO shows the best photocatalytic activity for (001)-Zn and (00$\overline{1}$)-O surface are easier to be attacked by holes resulting to lower quantum yield [31]. This finding was consistent with our experimental results, as ZnO 1D NNS nanostructures possessing the highest ratio of (101) facet (Additional file 1: Table S1).

The mechanism of photodegradation was described in Fig. 5d. Under UV irradiation, the photoexcitation of ZnO semiconductor generated large amounts of electron–hole pairs on the surface of catalyst. Band positions of the electrons (e^−^) in the CB and holes (h^+^) in the VB are reported as − 0.375 V and 2.875 V (vs. NHE), respectively [32]. While HOMO/LUMO value of the organic dye MO is − 0.036/1.644 V versus NHE [33]. The high oxidative potential of the holes permitted the direct oxidation of MO to reactive intermediates [34]. Furthermore, the photoinduced electron can be trapped by O_2_ and H_2_O molecules in the medium to generate highly active peroxide (O_2_^−·^) and hydroxyl radicals (·OH), respectively [20, 35–40]. All of these contributed to enhanced conductivity of H_2_O molecules around the catalyst and constituting a photocatalytic layer. The generated radicals attacked the organic MO molecules and oxidize them to intermediate by-products, which were partial mineralized to CO_2_ and H_2_O [41]. Different scavengers were added to survey the photocatalytic pathway and ascertain the main reactive species by trapping the holes and radicals in the photocatalytic reaction. A series of radical trapping experiments have been carried out under analogous conditions excepting of adding EDTA-2Na, BQ, and IPA into the reaction solution as scavenger of holes, O_2_^−·^ radicals and ·OH radicals, respectively. Figure 5e shows that the addition of EDTA-2Na and BQ strongly suppressed the photocatalytic activity, indicating that both of holes, O_2_^−·^ radicals were involved in the photocatalytic reaction. By contrast, the photocatalytic activity in ZnO 1D NNS could not be significantly suppressed by adding IPA, indicating that the mechanism might not involve the ·OH radicals. The highest suppression was found in EDTA-2Na, suggesting that the holes were highly involved in the mechanism of photocatalysis. Stability and reusability of photocatalyst is important for practical utility by reducing the overall cost. To evaluate the photostability of ZnO 1D NNS, the experiments of MO photodegradation procedure were conducted by recycling the reactions for five times. As shown in Fig. 5f, no noticeable loss of the photocatalytic activity was observed for MO degradation reaction after five recycles. Furthermore, the micromorphology and crystal change of ZnO 1D NNS before and after use in the photocatalytic degradation of MO is shown in Additional file 1: Fig. S5. The 1D NNS morphology is robust to be reserved after the recycled process along with partly fused beads of ZnO nanoparticles. Furthermore, the XRD pattern shows no apparent changes after the five recycles. The results confirm the stability and reusability of the ZnO 1D NNS photocatalyst.

## Conclusions

In summary, we developed an “anion-regulated morphology” strategy to achieve simple synthesis of 1D ZnO nanostructures. Anions of zinc precursors, playing an important role in the process of crystallization and shape transformation, could determine the final nanostructure by adjusting the surface energy of different facets in the ZnO seed. The acquired ZnO 1D NNS, benefiting from their dimensionality-generated uniform carrier transport, exhibited excellent photocatalytic performance, which could degrade MO efficiently under ultraviolet light irradiation. The scavenging experiment further proved that the photocatalytic process of ZnO catalyst was mainly controlled by reactive holes and O_2_^−·^ radicals. Our work provides new perspectives for the simple fabrication of 1D materials for further applications to photocatalysis, optoelectronic devices and energy harvesting.

## Supplementary information

**Additional file 1.** Spectroscopic investigation of ZnO nanostructures, morphological and crystal structural investigation of ZnO nanostructures to investigate the growth mechanism, the basis for quantitative analysis of MO in the photodegradation, and the stability of the ZnO photocatalyst.

## Data Availability

The following data are available in Additional file 1, Additional file 1: Fig. S1: UV–Vis spectra of the ZnO 1D NNS, nanoflowers, nanoflakes, nanoplates obtained by using zinc nitrate, zinc sulfate, and zinc chlorion as precursor. Additional file 1: Fig. S2: SEM images of ZnO nanorods prepared using zinc acetylacetonate as the precursor. Additional file 1: Fig. S3: SEM images of ZnO nanoflowers prepared by zinc nitrate exhibiting the grainy surface. Additional file 1: Fig. S4: The linear relationship between maximum absorbance and concentration of MO. Additional file 1: Fig. S5: SEM images and XRD patterns of ZnO 1D NNS before and after five recycles. Additional file 1: Table S1: The ratio of peak area (*A*_101_/*A*_002_) calculated from the XRD patterns of ZnO nanostructures. The conclusions made in this manuscript are based on the data which are all presented and shown in this paper.

## Abbreviations

1D: One-dimensional
NNS: Necklace-like nanostructures
DFT: Density functional theory
MO: Methyl orange
EDTA-2Na: Disodium ethylenediaminetetraacetate
BQ: 1,4-Benzoquinone
IPA: Isopropyl alcohol
SEM: Scanning electron microscope
TEM: Transmission electron microscopy
HRTEM: High-resolution TEM
XRD: X-ray powder diffraction
BET: Brunauer–Emmett–Teller
TOC: Total organic carbon
DFT: Density functional theory
GGA: Generalized gradient approximation
*C*_MO_: Concentration of MO
R: Degradation rate
A: Absorbance
*C*_0_: Initial concentration
*C*_t_: Concentration of the dye after irradiation time *t*
VB: Valence band
CB: Conduction band
Δ*E*: Difference of facet energy
