# Supplementary material for: Anion-Regulated Synthesis of ZnO 1D Necklace-Like Nanostructures with High Photocatalytic Activity
Source: Nanoscale Res Lett. 2020 Nov 4;15:206. doi: 10.1186/s11671-020-03435-5 (PMC7642098; doi:10.1186/s11671-020-03435-5)
Supplement: Supplementary file 1 — Additional file 1. Spectroscopic investigation of ZnO nanostructures, morphological and crystal structural investigation of ZnO nanostructures to investigate the growth mechanism, the basis for quantitative analysis of MO in the photodegradation, and the stability of the ZnO photocatalyst. [file 11671_2020_3435_MOESM1_ESM.docx]

**Supplementary Information**

Anions Regulated Synthesis of ZnO 1D Necklace-like Nanostructures with High Photocatalytic Activity

Xiaoyun Qin^1†^, Dongdong Shi^2†^, Bowen Guo^2†^, Cuicui Fu^3^, Jin Zhang^1^, Qingqing Xie^2^, Xiangdong Shi^1^, Fenghua Chen^1^, Xiaomei Qin^1^, Wei Yu^4^, Xiangli Feng^5^*, Yan Liu^3^*, and Dan Luo^2^*

^1^ School of Material and Chemical Engineering, Zhengzhou University of Light Industry, Zhengzhou 450002, China

^2^ State Key Laboratory of Heavy Oil Processing, College of New Energy and Materials, Beijing Key Laboratory of Biogas Upgrading Utilization, China University of Petroleum Beijing, Beijing 102249, China

^3^ Laboratory of Biomimetic Nanomaterials, Department of Orthodontics, Peking University School and Hospital of Stomatology, National Engineering Laboratory for Digital and Material Technology of Stomatology, Beijing Key Laboratory of Digital Stomatology, Beijing 100081, China

^4^ Key Lab of Separation Science for Analytical Chemistry, Dalian Institute of Chemical Physics, Chinese Academy of Sciences, Dalian, Liaoning 116023, China

^5^ Department of Stomatology, Hubei Provincial Hospital of Traditional Chinese Medicine, Wuhan 430065, China

^†^ X.Q., D.S. and B.G. contributed equally to this work.

***** Correspondence: doctorfengxiangli@163.com (X.F.); orthoyan@bjmu.edu.cn (Y.L.); luodan@cup.edu.cn (D.L.)

Received: date; Accepted: date; Published: date

**
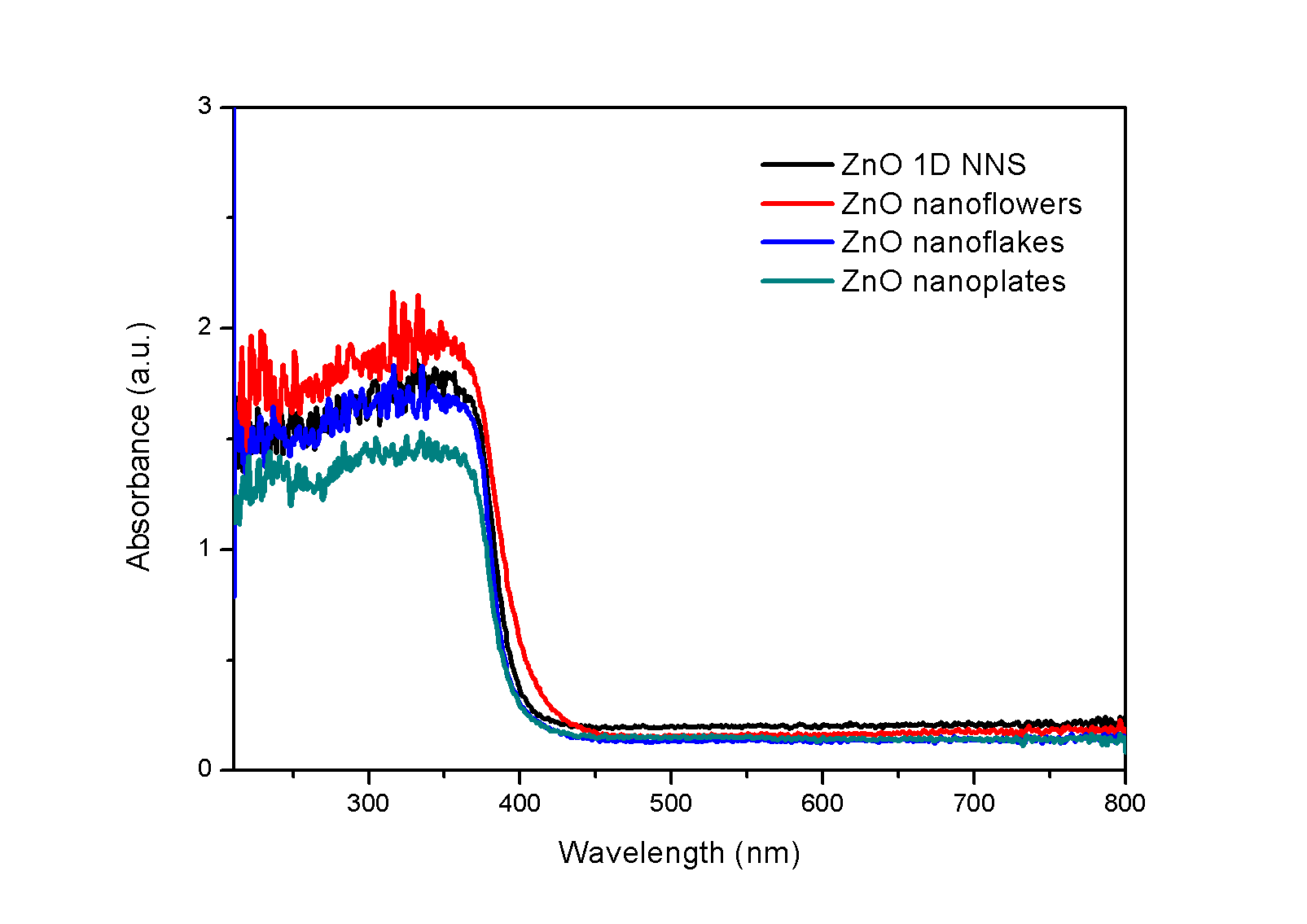
**

**Fig. S1** UV-vis spectra of the ZnO 1D NNS, nanoflowers, nanoflakes, nanoplates obtained by using zinc nitrate, zinc sulfate, and zinc chlorion as precursor.


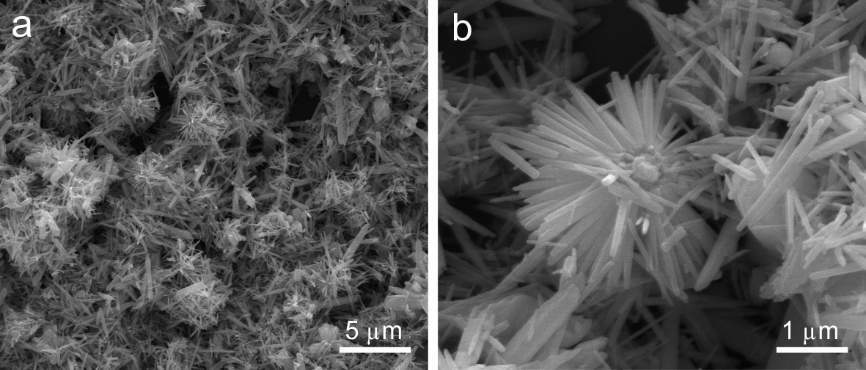


**Fig. S2** SEM images of ZnO nanorods prepared using zinc acetylacetonate as the precursor.


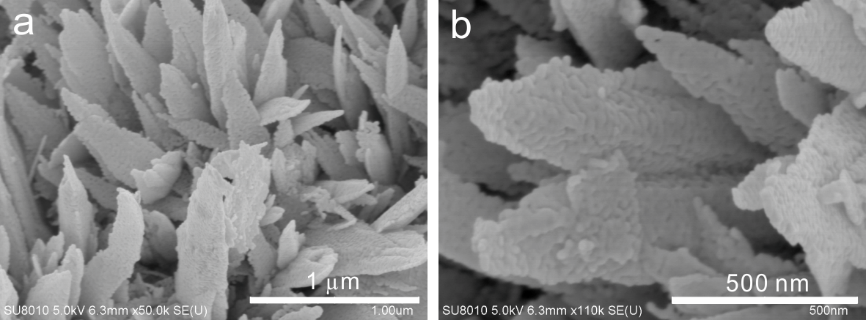


**Fig. S3** SEM images of ZnO nanoflowers prepared using zinc nitrate as the precursor exhibiting the grainy surface.


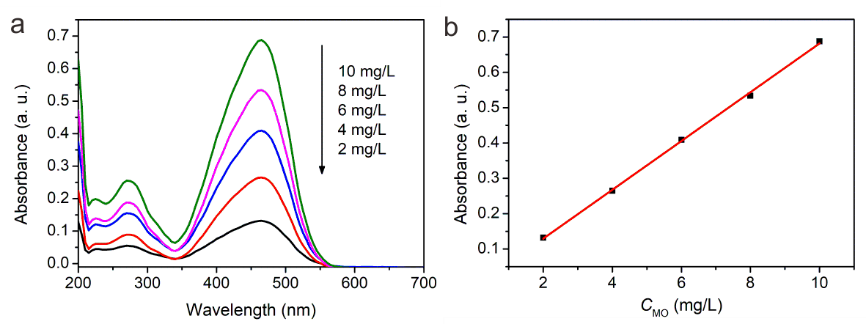


**Fig. S4** The UV-vis absorption curves of MO aqueous solution from low to high concentration.


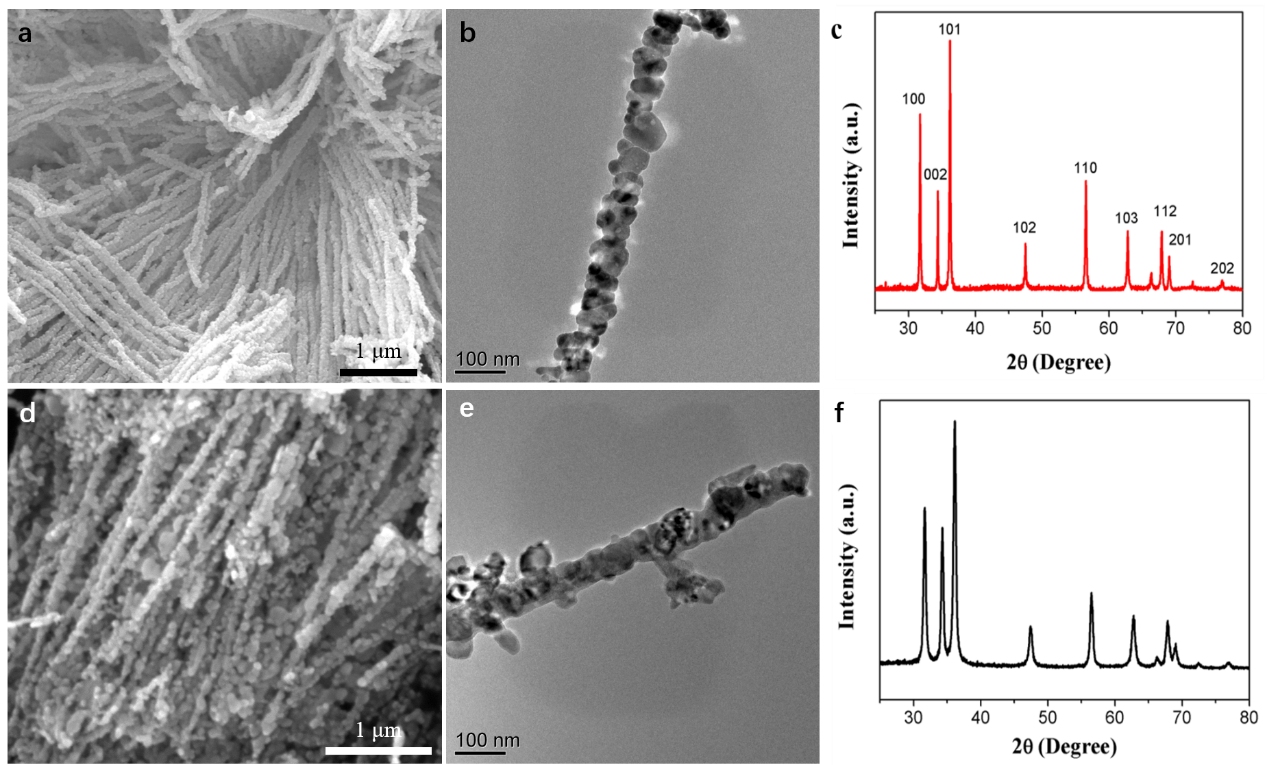


**Fig. S5** SEM, TEM images and XRD patterns of ZnO 1D NNS (a, b, c) before and (d, e, f) after five recycles.

**Table S1** The peak area and full width at half maximum (FWHM) calculated from the XRD patterns of ZnO nanostructures.

|  | *A*_101_ | fwhm_101_ | *a*_002_ | fwhm_002_ | *A*_101/_*a*_002_ |
| --- | --- | --- | --- | --- | --- |
| 1D NNS | 82777 | 0.311 | 37240 | 0.250 | 2.22 |
| nanoflowers | 55393 | 0.288 | 27200 | 0.242 | 1.71 |
| irregular nanoflakes | 48466 | 0.227 | 29246 | 0.234 | 1.65 |
| hexagonal nanoplates | 60256 | 0.465 | 56573 | 0.424 | 1.06 |
